# Detection of human herpesvirus 8 by quantitative polymerase chain reaction: development and standardisation of methods

**DOI:** 10.1186/1471-2334-12-210

**Published:** 2012-09-11

**Authors:** David J Speicher, Newell W Johnson

**Affiliations:** 1 School of Dentistry and Oral Health, Griffith University, Queensland, Australia; 2 Molecular Basis of Disease Research Program, Griffith Health Institute, Griffith University, Queensland, Australia; 3 Population & Social Health Research Program, Griffith Health Institute, Griffith University, Queensland, Australia

**Keywords:** HHV-8, Kaposi’s sarcoma, Human herpesvirus 8, Molecular diagnostics, Laboratory establishment

## Abstract

**Background:**

Human herpesvirus 8 (HHV-8), the aetiological agent of Kaposi’s sarcoma (KS), multicentric Castleman’s disease (MCD), and primary effusion lymphoma (PEL) is rare in Australia, but endemic in Sub-Saharan Africa, parts of South-east Asia and Oceania. While the treatment of external KS lesions can be monitored by clinical observation, the internal lesions of KS, MCD and PEL require extensive and expensive internal imaging, or autopsy. In patients with MCD and PEL, if HHV-8 viraemia is not reduced quickly, ~50% die within 24 months. HHV-8 qPCR is a valuable tool for monitoring HHV-8 viraemia, but is not available in many parts of the world, including those with high prevalence of KS and HHV-8.

**Methods:**

A new molecular facility with stringent three-phase workflow was established, adhering to NPAAC and CLSI guidelines. Three fully validated quantitative assays were developed: two for detection and quantification of HHV-8; one for GAPDH, necessary for normalisation of viral loads in tissue and peripheral blood.

**Results:**

The HHV-8 ORF73 and ORF26 qPCR assays were 100% specific. All qPCR assays, displayed a broad dynamic range (10^2^ to 10^10^ copies/μL TE Buffer) with a limit of detection of 4.85x10^3^, 5.61x10^2^, and 2.59x10^2^ copies/μL TE Buffer and a limit of quantification of 4.85x10^3^, 3.01x10^2^, and 1.38x10^2^ copies/μL TE Buffer for HHV-8 ORF73, HHV-8 ORF26, and GAPDH respectively.

The assays were tested on a panel of 35 KS biopsies from Queensland. All were HHV-8 qPCR positive with average viral load of 2.96x10^5^ HHV-8 copies/μL DNA extract (range: 4.37x10^3^ to 1.47x10^6^ copies/μL DNA extract): When normalised these equate to an average viral load of 2.44x10^4^ HHV-8 copies/10^3^ cells (range: 2.20x10^2^ to 7.38x10^5^ HHV-8 copies/10^3^ cells).

**Conclusions:**

These are the first fully optimised, validated and MIQE compliant HHV-8 qPCR assays established in Australia. They worked well for qualitative detection of HHV-8 in archival tissue, and are well-suited for quantitative detection in whole blood. They are now available for research, for clinical diagnosis of HHV-8 infection, and for monitoring treatment efficacy.

## Background

Human herpesvirus 8 (HHV-8), the aetiological agent of Kaposi’s sarcoma (KS), an AIDS-defining condition, multicentric Castleman’s disease (MCD) and primary effusion lymphoma (PEL) has been extensively characterized worldwide, but largely overlooked in Australia as the introduction of antiretroviral therapy in 1995–96 greatly reduced the incidence of KS. Australian researchers have looked briefly at the risk factors associated with the sexual transmission of HHV-8 [1,2], at detection methods [3,4], and at the genotyping of a few HHV-8 isolates [5]. Despite the low prevalence of HHV-8 associated disease in Australia, since 2007, 25 new HHV-8 PCR positive patients have been identified in Queensland, including one child aged just 3 years. In 2011, the first case of KS for 10 years was recognised at the Gold Coast Sexual Health Clinic, and a further eight cases of HHV-8 disease were also recognised clinically in the Brisbane area suggesting the possible re-emergence of HHV-8-related diseases and/or increased clinical recognition and testing. In Australia, most major anatomical pathology laboratories perform HHV-8 immunohistochemistry, but validated molecular assays are uncommon. HHV-8 nested PCR is performed at PathWest Laboratory Medicine, QEII Hospital, in Perth, Western Australia (the referral laboratory for Queensland Health), while real-time PCR (rtPCR) is performed at the Victorian Infectious Diseases Reference Laboratory (VIDRL) in Melbourne, Victoria and the South Eastern Area Laboratory Services (SEALS), Prince of Wales Hospital in Sydney, New South Wales. None of these laboratories perform quantitative PCR (qPCR) to monitor viral loads and treatment efficacy: This is of concern because, while the treatment of superficial KS can be monitored by clinical observation, both MCD and PEL require more extensive and expensive internal imaging, notably with computed axial tomography (CAT), magnetic resonance imaging (MRI) or positron emission tomography (PET) scans.

As the first research laboratory in Australia specialising in the detection and characterisation of HHV-8, our first objective was to establish fully validated HHV-8 qPCR assays to aid in the cost-effective and timely diagnosis of HHV-8 associated diseases, and to accurately monitor treatment regimes and save costs of other complex monitoring methods. Even with the high sensitivity of PCR, most asymptomatic patients are HHV-8 negative in this test, making PCR an ideal technique to monitor current infections [6-8]. This report provides details to assist with overcoming many of the challenges associated with establishing a new quantitative assay including: laboratory establishment, choice of positive and negative controls, and assay optimisation and validation for HHV-8 ORF73, HHV-8 ORF26, and the human reference gene glyceraldehyde 3-phosphate dehydrogenase (GAPDH) qPCR. These assays were used in an attempt to quantify HHV-8 in lesional biopsies but, due to issues with normalisation, are better suited for viral detection and monitoring of treatment by detecting and quantifying HHV-8 viraemia in peripheral blood mononuclear cells (PBMC).

## Methods

### Body cavity-based lymphoma (BCBL)-1 cell line harbouring latent HHV-8

BCBL-1 cells, an HHV-8 positive EBV negative cell line, were seeded at 3-5x10^5^ cells/mL (counted using trypan blue), and cultured in GIBCO® RPMI-1640 supplemented with 2 mM GlutaMAX™-I and 25 mM HEPES (Life Technologies™, Australia), as well as 10% HIA-FBS, 55 μM 2-mecaptoethanol, 1 mM sodium pyruvate, and 100 mM penicillin and streptomycin at 37°C in 5% CO_2_ in a humidified atmosphere, and passaged 50% every 3–4 days. Cell concentrations were maintained between 3x10^5^ cells/mL and 1x10^6^ cells/mL. Cell stocks were kept frozen in liquid nitrogen in 10% DMSO and 90% HIA-FBS, and were Mycoplasma free as determined by the PCR Mycoplasma Test Kit (AppliChem, USA). Viral and human genomic DNA were extracted from 100 μL of cultured cells suspension diluted 1:1 with PBS using the DNeasy Blood and Tissue Kit (Qiagen, Australia) following the manufacturer’s instructions, with a proteinase K digestion performed at 56°C for 10 minutes in a heating block. All samples were eluted in 100 μL of Buffer AE to increase the final DNA concentration.

### Cloning of HHV-8 and GAPDH constructs for qPCR standards

Amplicon containing –TA overhangs were produced by conventional PCR performed in a 20 μL volume containing 10X ThermoPol Reaction Buffer (New England BioLabs® Inc., Australia), 0.4 mM deoxyribonucleotide triphosphate (dNTP), 0.2 μM primers (GeneWorks, Australia), 0.24 U/μL Taq DNA polymerase (New England BioLabs® Inc., Australia) and 2 μL of nucleic acid extract (DNeasy Blood and Tissue Kit, Qiagen, Australia). The HHV-8 and GAPDH targets were amplified from DNA purified from BCBL-1 cells. PCR consisted of 45 cycles of amplification (20 seconds at 95°C; 30 seconds at 58°C, 30 seconds at 72°C), followed by a 15 minute extension step at 72°C in an iCycler Thermal Cycler (Bio-Rad, Australia). The entire 20 μL reaction was mixed with loading buffer (MO BIO Laboratories, Inc., Australia) and placed in a well of 2% w/v agarose low EEO (AppliChem, USA) containing SYBR® Safe DNA Gel Stain (10,000X concentrate in DMSO; Life Technologies™, Australia) in TAE buffer (0.04 M Tris-acetate, 1 mM EDTA) and analysed by agarose gel electrophoresis. A molecular weight marker (DMW-100M, GeneWorks, Australia) was added as a reference. Agarose gels were run at 120 V for 30 minutes, visualised by exposure to blue light (VersaDoc™ Imaging System 4000 MP, Bio-Rad, Australia) and examined with Quality One® V.6.3 1-D Analysis Software (Bio-Rad, Australia). Amplicon of the correct molecular weight was gel purified (QIAquick Gel Extraction Kit, Qiagen, Australia) and the eluted DNA quantified on the NanoDrop® ND-1000 Spectrophotometer (ThermoFisher Scientific, Australia).

Plasmid constructs were formed by ligating purified PCR amplicon into pGEM®-T Easy (Promega, USA) at a concentration of 3:1 respectively. The ligation reaction contained 2X Quick Ligation Reaction buffer and Quick T4 DNA Ligase (New England BioLabs® Inc., Australia) and was performed in a 20 μL volume at 25°C for 5 minutes. The construct was concentrated and salts were removed by mixing 1 mL butanol with 10 μL ligation mixture and then centrifuging at 14,000 *x g* for one minute, decanting the butanol and then drying in a Concentrator Plus (Eppendorf South Pacific, Australia) for 5 minutes. The construct was resuspended in 4 μL of dH_2_O, mixed with 30 μL of competent DH5α *Escherichia coli*, and incubated on ice for 5 minutes. Transformation was performed in a 2 mm cuvette by electroporation at 2.5 KV, 200 Ω, and 25 μF with the Gene Pulser Xcell™ System (Bio-Rad, Australia). Transformants were slowly shaken while incubated at 37°C for 40 minutes in 500 μL SOC broth (2% tryptone, 0.5% yeast extract, 10 mM NaCl, 2.5 mM KCl, 10 mM MgCl_2_, 20 mM glucose), plated out on 2YT agar containing 50 μg/mL ampicillin, and then grown overnight at 37°C. The resulting constructs were named following the convention pGEM®-T/XXXX, with the XXXX corresponding to the HHV-8 and human reference gene such as pGEM®-T/ORF73.

Colonies with constructs containing the gene of interest (GOI) were selected by colony PCR screening of the white colonies as follows: constructs were released from bacterial cells by boiling for 2 minutes in 15 μL dH_2_O and then examined by PCR using the M-13 universal sequencing primers (USP) [5^′^-GTAAAACGACGGCCAGT-3^′^ and reverse sequencing primers (RSP) [5^′^-ATTTCACACAGGAAACAGCTATGAC-3^′^[9]. PCR was subsequently performed in a 20 μL volume containing 10X ThermoPol Reaction Buffer (New England BioLabs® Inc., Australia), 0.4 mM dNTP, 0.2 μM primers (GeneWorks, Australia), 0.24 U/μL *Taq* DNA polymerase (New England BioLabs® Inc., Australia). Amplicon were produced by conventional PCR consisting of 25 cycles of amplification (60 seconds at 94°C; 90 seconds at 42°C; 60 seconds at 72°C) and then examined by agarose gel electrophoresis as previously described.

Colonies containing the desired construct were grown overnight at 37°C in 3 mL of 2YT broth containing 50 μg/mL ampicillin and catalogued as glycerol stocks (800 μL culture in 200 μL 80% glycerol). Plasmid DNA was extracted from the cultures with FastPlasmid™ Mini Kit (5 PRIME, Australia), their concentration determined by NanoDrop and diluted to 30 ng/μL. Constructs were catalogued at −80°C to minimise damage from freeze/thaw cycles, but working stocks were stored at 4°C [10]. Nucleotide sequencing was performed on both strands of the GOI within the pGEM®-T Easy using the ABI PRISM™ BigDye cycle sequencing kit (Perkin Elmer Applied Biosystems Division, USA) and the Applied Biosystems 3130xl capillary electrophoresis genetic analyser (Perkin Elmer Applied Biosystems Division, USA) at the Griffith University DNA Sequencing Facility (GUDSF, Griffith University (NATA accreditation #14814)). Nucleotide sequence alignments were analysed using BioEdit Sequence Alignment Editor® v7.0.5.2 [11] against known sequences available in GenBank.

### Optimisation and validation of quantitative PCR assays

All qPCR assays were fully optimised and validated for the detection of HHV-8 as outlined by the Australian National Pathology Accreditation Advisory Council (NPAAC), Clinical and Laboratory Standards Institute (CLSI), [formerly known as the National Committee for Clinical Laboratory Standards (NCCLS)] and by the Minimum Information for Publication of Quantitative Real-Time PCR Experiments (MIQE) Guidelines, using both cloned constructs and DNA extracted from BCBL-1 cells as the reference material [10,12-14]. The optimal primer annealing temperature (T_M_) was determined on BCBL-1 DNA extracts in duplicate with a temperature gradient from 55.0°C to 63.0°C on an iQ5 Real-Time PCR Detection System (Bio-Rad, Australia) using the QuantiFast SYBR Green PCR Kit (Qiagen, Australia). The primer concentration was determined in duplicate using a primer matrix on both sense and anti-sense primers so that the final concentration of the primers in the reaction mixture are tested from 50 nM to 900 nM [15,16]. The concentrations of the dual-labelled hydrolysis probes were determined by testing a range between 25 nM and 300 nM. The probes were tested for integrity by running negative controls of just water and probe at low and high probe concentrations. The optimal T_M_, primer concentration, and dual-labelled hydrolysis probe concentrations were determined by the amplification curve with the best shape and lowest cycle of quantification (Cq).

The accuracy, imprecision, PCR efficiency, linearity, measurable range, and repeatability, of the qPCR assays were validated using calibration curves [10-fold serial dilutions of the cloned constructs in TE Buffer (prepared in-house: 10 mM Tris–HCl, 1 mM EDTA, pH 8.0)]. Calibration curves were prepared fresh and examined in triplicate by qPCR on a Rotor-Gene Q (Qiagen, Australia). The actual copy number, determined by qPCR (y-axis), was compared to the calculated copy number (x-axis) graphically on Microsoft Excel with the standard deviations shown in vertical error bars and examined by linear regression analysis for linearity (R^2^), slope, intercept, and standard deviation of the residuals [17,18]. The calculated copy number was determined from the plasmid concentration determined via NanoDrop with the following formulae:

1. Molecular Weight (MW) (plasmid) in g/mol = plasmid length (bp) x 660 Da

2. Plasmid (copies/g) = Avogadro’s Number (6.02x10^23^)/MW(plasmid)

3. [Plasmid] in mol/L = [plasmid] as per NanoDrop (g/L) x MW (plasmid)

4. Plasmid (copies/μL) = [plasmid] mol/μL x MW (plasmid) x Plasmid (copies/g)

The calibration curve was rejected if the qPCR efficiency was outside the range of 95-105% (5% variance from 100%) and linearity (R^2^) was <0.9800. The measurable range of detection was determined by the linear region of the standard curve. The repeatability of the assay was measured by running the standard curve in triplicate over a 20 day period and comparing the copy numbers. The analytical sensitivity, determined as the limit of detection (LOD), and the limit of quantification (LOQ), were determined from a calibration curve serially diluted 2-fold. These calibration curves were produced in triplicate with the quantitative standard of highest concentration diluted from the quantitative standard of the calibration curve serially diluted 10-fold, which was 10-fold greater than the minimum detectible dilution. Each standard was examined three times per day for eight days [19]. The LOD was determined at the 95% confidence interval (CI) as being the point where no less than 23/24 samples were positive, whereas the LOQ was determined as the lowest concentration where the actual and calculated concentrations were nearly identical [19,20]. Since this project was performed on a single PCR machine by one operator, the robustness of the assay, although desired [21], could not be determined. Additionally, it would be ideal to include a comparative evaluation of this method against reference methods [10], but no such method is available in Australia.

The specificity of the HHV-8 qPCR assays were determined by running the assays against a panel of all HHV and were deemed specific if the assay only amplified the HHV-8 sample.

### Quantitative PCR assays

qPCR assays were designed to amplify a 142 bp and a 234 bp fragment of the HHV-8 ORF73 and ORF26 respectively as well as a 104 bp fragment of GAPDH from previously published primer and dual-labelled hydrolysis probe sequences (Table 1) [6,22,23]. qPCR assays were set up manually in 0.1 mL strip tubes (Qiagen, Australia; Cat#:981103) in a 20 μL volume using the QuantiFast™ Probe PCR + ROX Vial Kit (Qiagen, Australia) (containing HotStarTaq® *Plus* DNA Polymerase, dNTP mix, and QuantiFast™ PCR buffer), 0.2 μM primers, 0.1 mM dual-labelled hydrolysis probe (Purity: HPLC; GeneWorks, Australia), and 2 μL of nucleic acid extract. The qPCR reaction was performed on a Rotor-Gene Q starting with a five minute polymerase activation step at 95°C, followed by 45 cycles of amplification (5 seconds denaturation at 95°C; 30 seconds annealing/extension at 58°C). qPCR results were reported in “copies/μL TE Buffer” for the calibration curves and “copies/μL DNA extract” for the clinical isolates. For clinical samples the average viral loads were determined from both the HHV-8 ORF73 and HHV-8 ORF26 qPCR assays and then normalized to cell copy number, based on GAPDH results (there are two copies of GAPDH/cell) and then reported as HHV-8 copies/10^3^ cells. 

**Table 1:** Primers and probes used for HHV-8 and GAPDH qPCR assays

**Assay**	**Primers/Probe**	**Sequence (5**^**′**^**-3**^**′**^**)**	Nucleotide position*	**Amplicon length**	**Reference**
**Quantitative PCR Assays for HHV-8**					
HHV-8 ORF73	HHV8_73_01.1	GGTGATGTTCTGAGTACATAGCGG	124,326 - 124,349	142 bp	Lallemand *et al.*, 2000
	HHV8_73_02.1	CCGAGGACGAAATGGAAGTG	124,467 - 124,448		
	HHV8_73_Pb1	FAM-ACAAATTGCCAGTAGCCCACCAGGAGA-BHQ1^ξ^	124,421 - 124,395		
HHV-8 ORF26	HHV8_26_01.1	AGCCGAAAGGATTCCACCATT	47,287 - 47,304	234 bp	Hammock *et al.*, 2005
	HHV8_26_02.1	TCCGTGTTGTCTACGTCCAGA	47,519 - 47,499		
	HHV8_26_Pb.3	FAM-TGCAGCAGYTGTTGGTGTACCACAT-BHQ1^ξ^	47,378 - 47,402		
**Quantitative PCR Assays for Normalisation to Cell Counts**					
GAPDH	GAPDH_01.1	GCTCCCTCTTTCTTTGCAGCAAT	7.800 - 7,822	104 bp	Asahi-Ozaki *et al.*, 2006
	GAPDH_02.1	TACCATGAGTCCTTCCACGATAC	7,903 - 7,881		
	GAPDH_Pb1	FAM-TCCTGCACCACCAACTGCTTAGCACC-BHQ1^ξ^	7.826 - 7,851		

*Nucleotide position was determined from the reference sequences [GenBank:NC_003409] for HHV-8 and [GenBank:NG_007073.2] for GAPDH.

^ξ^Probes had 5^′^ terminus labeled with a FAM (6-carboxy-fluorescein) fluorophore and 3^′^ terminus labeled with BHQ-1 (Black Hole Quencher^®^).

### Controls and sample population

Analytical positive controls, and a range of negative controls (analytical, no-template control (NTC) and clinically negative tissues) were included on each PCR run to ensure accuracy of results. BCBL-1 extracts at a concentration ~10^4^ (10-fold higher than the assay LOD) and a 10-fold standard curve run in singular were included in each run to ensure PCR efficiency, linearity and that Cq values were consistent between runs [10]. Negative controls on each run included one sample from an HHV-8 negative oral squamous cell carcinoma biopsy and two to three NTC samples per run, and positioned at the front, middle and end of each sample set [10]. A PCR run was discarded due to inaccuracy if any of the following criteria occurred: (i) PCR efficiency differed by 5% of the validated efficiency, (ii) the Cq value of the positive control differed by more than 1, (ii) any of the negative controls were positive so that a definite sample concentration could be determined, or if (iv) the standard curve was not linear (R^2^ <0.98000).

The assays were tested on an opportunistic collection of KS archival lesions from various Queensland hospitals retrospectively following ethical clearance (Griffith University Human Research Ethics Committee (HREC) (DOH/05/07/HREC), RBWH HREC (#2007/089), PAH HREC (#2007/117), Townsville Hospital HREC (#39/07), Gold Coast Hospital HREC (#200750), the Director-General of Queensland Health (BR033835, 000784–3), and Clinical and Statewide Services (#128)). The sample population consisted of 35 biopsies of KS lesions from 24 patients presenting between 2004 and 2011: Eight biopsies were from five HIV-negative patients (males:females 3:2) with a mean age of 83.05 years (range: 78.94-88.60 years) while 39 biopsies were from 31 HIV-positive males with a mean age of 43.76 years (range: 28.16-66.28 years).

All clinical samples were examined by both HHV-8 qPCR assays, to determine the viral load with accuracy, and to avoid the risk of false positives and negatives [10,24]. If a sample yielded a negative result it was re-examined with GAPDH to examine for inhibition, to determine that adequate DNA was in the sample and to verify that DNA had been extracted [10]. If a sample yielded a positive result but the concentration was below the LOD, it was repeated. If the repeated test showed the sample was negative or yielded a second positive result that was below the LOD, it was labelled as “not detected”. If a sample was strongly positive by one of the HHV-8 qPCR assays, but negative in the other assay the sample was repeated in triplicate with the other assay. If the repeat testing showed that the sample was repeatedly negative in only one of the assays then it was recorded, accordingly: e.g. HHV-8 ORF73 positive, HHV-8 ORF26 negative.

## Results and Discussion

Establishing an entirely new molecular laboratory is difficult, complex and time consuming: especially for work with viruses that are rare in a particular geographical area. Whether designed for research only or for clinical diagnostics, in order to produce quality results a molecular laboratory must have stringent workflows based on the NPAAC guidelines [10].

PCR contamination was minimized by implementing basic laboratory practices, chemical barriers, strict sterilization practices, and a strict unidirectional PCR workflow with three physically separate areas for: preparation of reagents; DNA extraction and template adding; and PCR amplification and post-PCR manipulations [10,25-27]. However, due to space constraints, DNA extraction was performed on a bench in the main laboratory with template added to the PCR reaction mixture in a Top-safe 1.2 biological safety cabinet class II (BSCII) (Bio-Cabinets Australia Pty, Australia). Each area contained its own dedicated equipment, reagents and personal protective equipment [10]. As rtPCR was performed as a sealed system, amplification was also performed in the main laboratory with the tubes only opened in the contained post-amplification area [10].

Carryover contamination was prevented by the strict implementation of the unidirectional flow from pre-amplification to post-amplification areas, with only sealed tubes and racks travelling down the workflow [10]. If anything needed to be re-used and go against the unidirectional flow, such as PCR racks or bottles, they were decontaminated overnight in a 1:100 dilution of Trigene® Advance (MediChem International Ltd, UK) before returning to the pre-amplification area [10]. Cross-contamination was prevented from all DNA extractions and template handling by wiping down the laboratory bench and BSCII with a 1:20 dilution of Trigene® Advance followed by UV radiation for 20 minutes. Trigene® Advance was used as a spray at 1:20 and 1:100 to soak used racks overnight due to its cost effectiveness and ability to degrade DNA.

All molecular assays require clinical and analytical, positive and negative controls. Obtaining positive control samples requires formal ethical clearance which can take many months. The usefulness of three types of molecular analytical positive controls were therefore reviewed: the uni-control method, synthetic controls (oligonucleotides and/or plasmids), and plasmids (imported or in-house) cloned from clinical isolates/cell lines (Table 2). Both the uni-control method [28] and the production of synthetic controls [29] are good options, especially for the detection of rare or hazardous viruses, as their simplistic nature produces the desirable amplification target without the need for infectious virus. The uni-control method is a quick and simple procedure that attaches primer binding sites to the ends of PCR amplicon, but it is only an interim solution until wild-type material is available, and it cannot be used to determine viral loads or to detect low viral loads. The production of synthetic oligonucleotides, whole genes or viral open reading frames can be used for quantitative PCR assays, but can be expensive. While most primer/probe manufacturing companies can produce synthetic oligonucleotides these cannot be quantified, and the amplicon must be small (≤200 bp). As neither of these methods were ideal for our assays, nor are HHV-8 quantitative controls available within Australia, an attempt was made to import plasmids from international laboratories. While this can be cost effective, after facing difficulties with the Australian Quarantine and Inspection Service (AQIS) and several failed attempts to clone the plasmids, we acquired an HHV-8 positive, EBV-negative body-cavity-based lymphoma cell line BCBL-1 [30]. These are freely available from the NIH AIDS Research and Reference Reagent Program, easy to propagate, and do not require ethical clearance. However, producing constructs via cloning has several inherent risks, especially cross-contamination due to the high copy numbers present: these were avoided by the strict unidirectional workflow. 

**Table 2:** Advantages and disadvantages of three types of molecular analytical positive controls

**Positive control**		**Advantages**	**Disadvantages**	**Reference**
Uni-Control Method		• Can be used in the absence of wild-type control material	• Qualitative only not quantitative	Whiley et al., 2010
		• Reduced contamination	• Cannot be used for low viral loads	
		• DNA and RNA compatible	• Greater risk of primer dimer formation	
		• Quick and simple method	• Interim solution until wild-type material is available	
			• Does not account for sequence variation in clinical material	
Synthetic Controls		• DNA and RNA compatible	• Synthetic oligonucleotides must be less than 200 bp	Smith et al., 2006
(Oligonucleotides and/or Plasmids)		• Reduced contamination (does not require cloning)	• Can be expensive if target is large	
		• Production of a synthetic oligonucleotide and clone into a plasmid	• Target sequence must be known	
		• No possibility of producing false-positives	• Requires two separate control reactions (primer and probe)	
		• Can be used for rtPCR and conventional PCR		
Cloned Plasmids	Imported	• Pre-made available (other labs or PlasmID repository)	• Requires shipping	None
			• Difficult to distinguish contamination from clinical material	
		• Cost effective		
		• Can be quantitative and qualitative	• Possible contamination	
	Produced “in-house”	• Can be quantitative and qualitative	• Requires clinical material or live virus	None
		• Cost effective	• Requires cloning (time consuming)	
		• Can easily be produced	• Difficult to distinguish contamination from clinical material	
			• Possible contamination	

All constructs were cloned from DNA purified from BCBL-1 cells, but diluted 1:1,000 in TE Buffer to yield a Cq between 20 and 25 for optimising primers and hydrolysis probes. The sequences of all pGEM-T/ORF73 [GenBank:JN613421] and pGEM-T/GAPDH [GenBank:JN613427-JN613429] constructs were identical to the published sequences [GenBank:NC_003409] and [GenBank:NG_007073.2] respectively (Additional file 1 and 2). The sequence of the pGEM-T/ORF26 construct [GenBank:JN613422-JN613426] had two conserved mismatches in all sequenced constructs; a C → T mismatch within the hydrolysis probe binding site: this required the probe sequence to be modified so that a pyrimidine (Y) would bind to both possible nucleotides (Additional file 3). The second mismatch was irrelevant. Using these constructs on a gradient block revealed the optimal T_M_ to be 58.0°C for the HHV-8 ORF73 and HHV-8 ORF26 rtPCR assays. The GAPDH rtPCR assay had two optimal T_M_, 58.0°C and 62.0°C: the lower temperature was used so that all three assays could be performed simultaneously. Using a primer concentration matrix and hydrolysis probe dilutions, the optimal concentrations were determined to be 200 nM of both sense and anti-sense primers and 100 nM of hydrolysis probe (Table 3).

**Table 3:** Optimised conditions and PCR dynamics

**Assay**	**HHV-8 ORF73**	**HHV-8 ORF26**	**GAPDH**
Primer T_M_ (°C)	58.0	58.0	58.0
[Primer]	200 nM, both primers	200 nM, both primers	200 nM, both primers
[Probe]	100 nM	100 nM	100 nM
Slope	0.96842 ± 0.02787	0.97243 ± 0.03028	0.99701 ± 0.02886
Intercept	0.27100 ± 0.20289	0.21521 ± 0.19569	0.02605 ± 0.19293
Linearity (R2)	0.99424	0.99230	0.99334
SD of Residuals	0.21584	0.27500	0.26213
Efficiency (%)	99.42	100.4	99.26
Linear Range	4.85E+03 to 6.34E+10	1.52E+02 to 1.95E+10	1.38E+02 to 3.44E+10
LOD	4.85E+03 ± 3.02E+03	5.61E+02 ± 5.46E+02	2.59E+02 ± 2.48E+02
LOQ	4.85E+03 ± 3.02E+03	3.01E+02 ± 2.43E+02	1.38E+02 ± 1.17E+02

**Figure 1:**
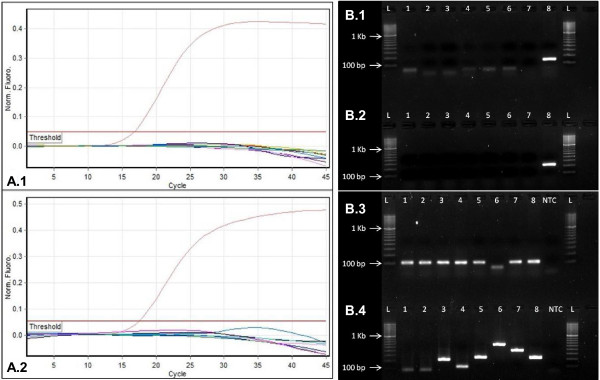
**Specificity of the HHV-8 ORF73 and ORF26 qPCR Assays.** Amplification curves (**A**) and agarose gel electrophoresis images (**B**) of the HHV-8 ORF73 (**1**) and ORF26 (**2**) assays tested against a panel of all eight HHV. The GAPDH PCR assay (**B.3**) was used as a DNA control as all samples were prepared from cell lines, except for HHV-6 (lane 6). Various primer sets were used to test HHV-1 to −8 (lanes 1 to 8 respectively). A 100 bp molecular weight marker was used as a reference.

Analytical specificity was tested in triplicate on a panel of DNA from all eight HHV. DNA was extracted from separate cultures of Vero cells infected with HSV-1 and HSV-2, cells carrying VZV, lymphoblastoid cell lines carrying EBV, SupT1 cells carrying HHV-7, and BCBL-1 carrying HHV-8. HCMV DNA was extracted from placental tissue known to be HCMV positive, and HHV-6 DNA was obtained from viral stocks. For all viruses, except HHV-8, the HHV-8 rtPCR assays yielded a negative result when examined via rtPCR and agarose gel electrophoresis, thus equating to an analytical specificity of 100% (Figure 1). This is unsurprising, considering the specificity of PCR and that all HHV-8 primer sequences were HHV-8 specific when analysed with NCBI’s nucleotide-nucleotide basic local alignment search tool (BLASTn). GAPDH amplified most samples of the specificity panel due the virion being propagated from human cell lines, except the HHV-6 sample for which the agarose gel image showed possible primer-dimer formation.

**Figure 2:**
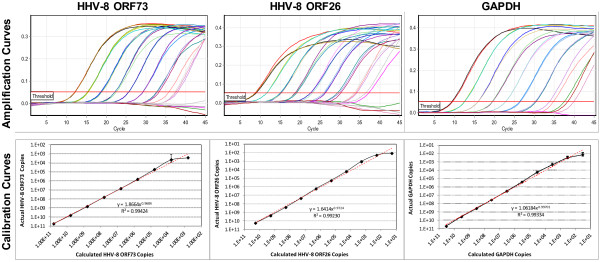
**Amplification and calibration curves.** Amplification and calibration curves used to determine the linear range and efficiency from a 10-fold serial dilution of cloned constructs.

**Figure 3:**
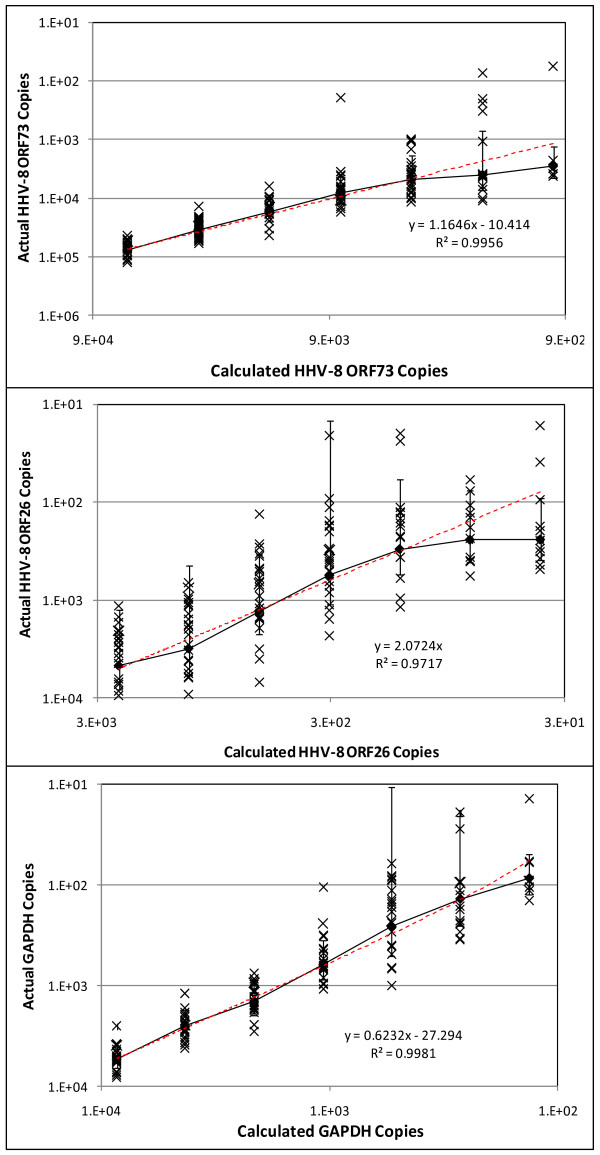
**Assay sensitivity.** Calibration curves using a ½-serial dilution of cloned constructs to determine the limit of detection and limit of quantification of the HHV-8 ORF73, HHV-8 ORF26, and GAPDH qPCR assays.

Calibration curves produced a linear relationship for all qPCR assays from 10^10^ down to 10^2^ copies/μL TE Buffer. Both HHV-8 and the GAPDH qPCR assays produced nearly perfect efficiency and linearity (R^2^) with values of 99.40% and 0.99424 for the HHV-8 ORF73 qPCR assay, 100.40% and 0.99230 for the HHV-8 ORF26 qPCR assay, and 99.26% and 0.99334 for the GAPDH qPCR assay (Figure 2; Table 3). Calibration curves with 2-fold serial dilutions displayed near perfect replicates from 6.43x10^4^ to 8.04x10^3^ copies/μL TE Buffer (average coefficient of variation (CV) = 0.4088) and from 8.60x10^3^ to 1.07x10^3^ copies/μL TE Buffer for the HHV-8 ORF73 and GAPDH assays respectively (Figure 3). The LOD and LOQ of the HHV-8 ORF73 qPCR assay were equivalent at 4.85x10^3^ ± 3.02x10^3^ copies/μL TE Buffer (Cq = 37.44 ± 1.05) whereas for the GAPDH qPCR assay the LOD was 2.59x10^2^ ± 2.48x10^2^ copies/μL TE Buffer (Cq = 37.25 ± 2.16) but the LOQ was 1.38x10^2^ ± 1.17x10^2^ copies/μL TE Buffer (Cq = 38.52 ± 2.70) (Table 3). The sensitivity (LOD and LOQ) of the HHV-8 ORF26 assay was 10-fold greater than the HHV-8 ORF73 qPCR assay but the CV was much higher (CV = 0.7740) with LOD of 5.61x10^2^ ± 5.46x10^2^ copies/μL TE Buffer (Cq = 35.51 ± 1.54) and LOQ of 3.01x10^2^ ± 2.43x10^2^ copies/μL TE Buffer (Cq = 36.78 ± 2.02) (Table 3).

Although the LOD of our HHV-8 ORF73 qPCR assay is 10 to 100-fold greater than previously reported using this primer set [6,22], scrutinising these previously published data suggests that the LOD given by these authors is not as sensitive as stated. The assay reported by Lallemand *et al.*[22] displayed a linear standard curve between 10^6^ to 10^1^ copies, but data for linearity and efficiency were not given: It can be assumed, however, that their assay was not linear above 10^5^ copies nor below 10^2^ copies, based on the amplification curves shown and the high CV values reported. Using the same primer and probe set, Asahi-Ozaki *et al.*[6] displayed a linear relationship from 10^8^ to 10^1^ copies for both the HHV-8 ORF73 and GAPDH assay, but close examination of their HHV-8 ORF73 amplification curves shows tight replicates down to 10^2^ copies: It is unlikely that the assay can yield accurate results below this level with any degree of accuracy. As our amplification curves appear identical to theirs for both assays it is most likely that the sensitivities of both our assays are comparable (Figure 2). As no HHV-8 ORF26 qPCR assay has been published, no such comparisons can be made for this.

The primer/probe sequences of our HHV-8 ORF26 qPCR assay have been used by many laboratories [23,31], but problems with false positives have been reported [32]. Hammock *et al.*[23] perform their PCR assays with up to 50 cycles in order to detect potentially very low viral loads. While many claims have been made regarding the nature of HHV-8 in lesional tissue based on samples amplifying after many cycles (i.e. >40 cycles), it is our experience that the LOD of the HHV-8 ORF26 assay is 5.61x10^2^ ± 5.46x10^2^ copies/μL TE Buffer (Cq = 35.51 ± 1.54) and thus any sample amplifying below this threshold is likely to be a false positive: claims based on data from large cycle numbers are not reliable. This is critical when used clinically, especially in patients with MCD whose disease cannot be easily monitored visually, for if HHV-8 viraemia is not reduced quickly the patient could be dead in 10 months [33].

In order to eliminate the risk of false positives, clinical isolates were tested with both the HHV-8 ORF73 and ORF26 assays [24]: these showed that all KS biopsies (35/35) were HHV-8 positive. The average viral load was 2.96x10^5^ HHV-8 copies/μL DNA extract (range: 4.37x10^3^ to 1.47x10^6^ HHV-8 copies/μL DNA extract), and when normalised equates to an average viral load of 2.44x10^4^ HHV-8 copies/10^3^ cells (range: 2.20x10^2^ to 7.38x10^5^ HHV-8 copies/10^3^ cells). While it was expected that all samples would be PCR positive, because the pathology reports stated the tissues were HHV-8 immunohistochemistry positive, no conclusion can be drawn regarding the viral loads in different stages of KS lesional tissue. Others have claimed to do so [6,31], but it is impossible to normalise to the precise number of virus infected cells in the tissue. These assays are, therefore, best restricted to qualitative detection of virus in tissue samples, although they can give qualitative results in peripheral blood where accurate cell counts can be made. Either way these assays are clearly a valuable tool for detection of HHV-8 infection in a range of clinical samples.

## Conclusions

Two fully optimised, validated, and MIQE compliant HHV-8 assays have been established with excellent sensitivity, specificity and dynamics. These assays can be normalised by cell counts based on GAPDH qPCR results for use in PBMC. These are the first HHV-8 qPCR assays established in Australia and are now available for both research and clinical diagnostics.

## Abbreviations

BCBL: Body-cavity-based lymphoma; BLASTN: Nucleotide-nucleotide basic local alignment search tool; BSCII: Biological safety cabinet class II; CAT: Computed Axial Tomography; CLSI: Clinical and Laboratory Standards Institute; Cq: Cycle of quantification; CV: Coefficient of variation; EBV: Epstein-Barr virus; GAPDH: Glyceraldehyde 3-phosphate dehydrogenase; GOI: Gene of interest; GUDSF: Griffith University DNA Sequencing Facility; HAART: Highly active antiretroviral therapy; HCMV: Human cytomegalovirus; HIA-FBS: Heat inactivated foetal bovine serum; HHV: Human herpesvirus; HSV: Herpes simplex virus; KS: Kaposi’s sarcoma; LOD: Limit of detection; LOQ: Limit of quantification; MCD: Multicentric Castleman’s disease; MIQE: Minimum Information for Publication of Quantitative Real-Time PCR Experiments; MRI: Magnetic resonance imaging; NCCLS: National Committee for Clinical Laboratory Standards; NPAAC: National Pathology Accreditation Advisory Council; NTC: No template control; ORF: Open reading frame; PBMC: Peripheral blood mononuclear cells; PCR: Polymerase chain reaction; PEL: Primary effusion lymphoma; PET: Positron emission tomography; QPCR: Quantitative PCR; RSP: Reverse sequencing primer; RtPCR: Real-time PCR; T_M_: Annealing temperature; USP: Universal sequencing primer; VIDRL: Victorian Infectious Diseases Reference Laboratory.

## Competing interests

The authors declare that they have no competing interests.

## Authors’ contributions

DJS was responsible for the development of the assays, acquisition and interpretation of data, and drafting the manuscript. NWJ initiated the HHV-8 research program, and contributed to manuscript revision and final presentation. All authors read and approved the final manuscript.

## Pre-publication history

The pre-publication history for this paper can be accessed here:

http://www.biomedcentral.com/1471-2334/12/210/prepub

## Supplementary Material

Additional file 1 **Multiple alignment of the pGEM-T/ORF73 constructs with reference, primers, and probe sequences.** Multiple alignment of pGEM-T/ORF73 construct sequence from DH5α E. coli colony #3 (c-3) and the HHV-8 ORF73 sense (HHV8_73_01.1) and anti-sense (HHV8_73_02.1) primers and hydrolysis probe (HHV8_73_Pb1) with the HHV-8 ORF73 [GenBank:NC_003409] with periods indicating identical nucleotide bases as the reference sequence. Click here for file

Additional file 2 **Multiple alignment of the pGEM-T/GAPDH constructs with reference, primers, and probe sequences.** Multiple alignment of pGEM-T/GAPDH construct sequence from three DH5α E. coli colonies (C-1 to C-3) and the HHV-8 ORF73 sense (GAPDH_01.1) and anti-sense (GAPDH_02.1) primers and hydrolysis probe (GAPDH_Pb1) with the reference sequence [GenBank:NG_007073.2] with periods indicating identical nucleotide bases as the reference sequence. Click here for file

Additional file 3 **Multiple alignment of the pGEM-T/ORF26 constructs with reference, primers, and probe sequences.** Multiple alignment of pGEM-T/ORF26 construct sequence from five DH5α E. coli colonies and the HHV-8 ORF26 sense (HHV8_26_01.1) and anti-sense (HHV8_26_02.1) primers and hydrolysis probe (HHV8_26_Pb1.1) with the HHV-8 ORF73 [GenBank:NC_003409] with periods indicating identical nucleotide bases as the reference sequence. Click here for file
