# Supplementary material for: Detection of human herpesvirus 8 by quantitative polymerase chain reaction: development and standardisation of methods
Source: BMC Infect Dis. 2012 Sep 11;12:210. doi: 10.1186/1471-2334-12-210 (PMC3490733; doi:10.1186/1471-2334-12-210)
Supplement: Additional file 1 — Multiple alignment of the pGEM-T/ORF73 constructs with reference, primers, and probe sequences. Multiple alignment of pGEM-T/ORF73 construct sequence from DH5α E. coli colony #3 (c-3) and the HHV-8 ORF73 sense (HHV8_73_01.1) and anti-sense (HHV8_73_02.1) primers and hydrolysis probe (HHV8_73_Pb1) with the HHV-8 ORF73 [GenBank:NC_003409] with periods indicating identical nucleotide bases as the reference sequence. [file 1471-2334-12-210-S1.pdf]

```
                            10        20        30        40        50
                   ....|....|....|....|....|....|....|....|....|....|
HHV-8 NC_003409    GTCTGTGGGGTGGTGATGTTCTGAGTACATAGCGGTATTCGCGAGATGGG
ORF73 C-3          CA..AGT.AT........................................
HHV8_73_01.1       -----------........................---------------
HHV8_73_02.1       --------------------------------------------------
HHV8_73_Pb1        --------------------------------------------------

                            60        70        80        90       100
                   ....|....|....|....|....|....|....|....|....|....|
HHV-8 NC_003409    CCAGGTTGTGGGTCATCGTCTGGTGTATTATCTCCTGGTGGGCTACTGGC
ORF73 C-3          ..................................................
HHV8_73_01.1       --------------------------------------------------
HHV8_73_02.1       --------------------------------------------------
HHV8_73_Pb1        ------------------------------....................

                           110       120       130       140       150
                   ....|....|....|....|....|....|....|....|....|....|
HHV-8 NC_003409    AATTTGTTCATGTGTGCTAACAACAGGGTAATCCACTTCCATTTCGTCCT
ORF73 C-3          ..................................................
HHV8_73_01.1       --------------------------------------------------
HHV8_73_02.1       ---------------------------------.................
HHV8_73_Pb1        .......-------------------------------------------

                           160       170
                   ....|....|....|....|.
HHV-8 NC_003409    CGGATGACGACCCGTGCAAGA
ORF73 C-3          ....ATCGA.TT.CC..GGCC
HHV8_73_01.1       ---------------------
HHV8_73_02.1       ...------------------
HHV8_73_Pb1        ---------------------
```
