# Supplementary material for: Detection of human herpesvirus 8 by quantitative polymerase chain reaction: development and standardisation of methods
Source: BMC Infect Dis. 2012 Sep 11;12:210. doi: 10.1186/1471-2334-12-210 (PMC3490733; doi:10.1186/1471-2334-12-210)
Supplement: Additional file 2 — Multiple alignment of the pGEM-T/GAPDH constructs with reference, primers, and probe sequences. Multiple alignment of pGEM-T/GAPDH construct sequence from three DH5α E. coli colonies (C-1 to C-3) and the HHV-8 ORF73 sense (GAPDH_01.1) and anti-sense (GAPDH_02.1) primers and hydrolysis probe (GAPDH_Pb1) with the reference sequence [GenBank:NG_007073.2] with periods indicating identical nucleotide bases as the reference sequence. [file 1471-2334-12-210-S2.pdf]

```
                            10        20        30        40        50        60
                    ....|....|....|....|....|....|....|....|....|....|....|....|
HHV-8 NC_003409     CAGTGCTACCCCCATTTTTTAGCCGAAAGGATTCCACCATTGTGCTCGAATCCAACGGAT
HHV8 ORF26 C-1      .G.-.AATT.------GA..........................................
HHV-8 ORF26 C-2     .G.-.AATT.------GA.............................A............
HHV-8 ORF26 C-3     .G.-.AATT.------GA..........................................
HHV-8 ORF26 C-4     .G.-.AATT.------GA..........................................
HHV-8 ORF26 C-5     .C.C.AATT.A.T.G.GA..........................................
HHV8_26_01.1        --------------------.....................-------------------
HHV8_26_02.1        ------------------------------------------------------------
HHV8_26_Pb1.1       ------------------------------------------------------------

                            70        80        90       100       110       120
                    ....|....|....|....|....|....|....|....|....|....|....|....|
HHV-8 NC_003409     TTGACCTCGTGTTCCCCATGGTCGTGCCGCAGCAACTGGGGCACGCTATTCTGCAGCAGC
HHV8 ORF26 C-1      ...........................................................T
HHV-8 ORF26 C-2     ...................................T.......................T
HHV-8 ORF26 C-3     ...........................................................T
HHV-8 ORF26 C-4     ...........................................................T
HHV-8 ORF26 C-5     ..........................................G................T
HHV8_26_01.1        ------------------------------------------------------------
HHV8_26_02.1        ------------------------------------------------------------
HHV8_26_Pb1.1       ---------------------------------------------------.........

                           130       140       150       160       170       180
                    ....|....|....|....|....|....|....|....|....|....|....|....|
HHV-8 NC_003409     TGTTGGTGTACCACATCTACTCCAAAATATCGGCCGGGGCCCCGGATGATGTAAATATGG
HHV8 ORF26 C-1      ....................................................C.......
HHV-8 ORF26 C-2     ....................................................C.......
HHV-8 ORF26 C-3     .....A..............................................C.......
HHV-8 ORF26 C-4     ....................................................C.......
HHV-8 ORF26 C-5     ....................................................C.......
HHV8_26_01.1        ------------------------------------------------------------
HHV8_26_02.1        ------------------------------------------------------------
HHV8_26_Pb1.1       ................--------------------------------------------

                           190       200       210       220       230       240
                    ....|....|....|....|....|....|....|....|....|....|....|....|
HHV-8 NC_003409     CGGAACTTGATCTATATACCACCAATGTGTCATTTATGGGGCGCACATATCGTCTGGACG
HHV8 ORF26 C-1      ............................................................
HHV-8 ORF26 C-2     ............................................................
HHV-8 ORF26 C-3     ...................................................A........
HHV-8 ORF26 C-4     .....................T.G....................................
HHV-8 ORF26 C-5     ............................................................
HHV8_26_01.1        ------------------------------------------------------------
HHV8_26_02.1        ----------------------------------------------------........
HHV8_26_Pb1.1       ------------------------------------------------------------

                           250       260       270
                    ....|....|....|....|....|....|.
HHV-8 NC_003409     TAGACAACACGGATCCACGTACTGCCCTGCG
HHV8 ORF26 C-1      .............AATCAC..G..AAT.CGC
HHV-8 ORF26 C-2     .............AATCAC..G..AAT.CGC
HHV-8 ORF26 C-3     .............AATCAC..G..AAT.CGC
HHV-8 ORF26 C-4     .............AATCAC..G..AAT.CGC
HHV-8 ORF26 C-5     .............AATC----GAATT.CCGC
HHV8_26_01.1        -------------------------------
HHV8_26_02.1        .............------------------
HHV8_26_Pb1.1       -------------------------------
```
