# Supplementary material for: Detection of human herpesvirus 8 by quantitative polymerase chain reaction: development and standardisation of methods
Source: BMC Infect Dis. 2012 Sep 11;12:210. doi: 10.1186/1471-2334-12-210 (PMC3490733; doi:10.1186/1471-2334-12-210)
Supplement: Additional file 3 — Multiple alignment of the pGEM-T/ORF26 constructs with reference, primers, and probe sequences. Multiple alignment of pGEM-T/ORF26 construct sequence from five DH5α E. coli colonies and the HHV-8 ORF26 sense (HHV8_26_01.1) and anti-sense (HHV8_26_02.1) primers and hydrolysis probe (HHV8_26_Pb1.1) with the HHV-8 ORF73 [GenBank:NC_003409] with periods indicating identical nucleotide bases as the reference sequence. [file 1471-2334-12-210-S3.pdf]

```
                             10        20        30        40        50
                    ....|....|....|....|....|....|....|....|....|....|
GAPDH NG_007073.2   ACTTGCGCCCCGCTCCCTCTTTCTTTGCAGCAATGCCTCCTGCACCACCA
GAPDH C-1           CAC.AGTGATT.......................................
GAPDH C-2           CAC.AGTGATT.......................................
GAPDH C-3           CAC.AGTGATT.......................................
GAPDH 01.1          -----------.......................----------------
GAPDH 02.1          --------------------------------------------------
GAPDH_Pb1           -------------------------------------.............

                             60        70        80        90       100
                    ....|....|....|....|....|....|....|....|....|....|
GAPDH NG_007073.2   ACTGCTTAGCACCCCTGGCCAAGGTCATCCATGACAACTTTGGTATCGTG
GAPDH C-1           ..................................................
GAPDH C-2           ..................................................
GAPDH C-3           ..................................................
GAPDH 01.1          --------------------------------------------------
GAPDH 02.1          ------------------------------------------........
GAPDH_Pb1           .............-------------------------------------

                            110       120       130
                    ....|....|....|....|....|....|
GAPDH NG_007073.2   GAAGGACTCATGGTATGAGAGCTGGGGAAT
GAPDH C-1           ...............AATCGAA.TCCCGCG
GAPDH C-2           ...............AATCGAA.TCCCGCG
GAPDH C-3           ...............AATCGAA.TCCCGCG
GAPDH 01.1          ------------------------------
GAPDH 02.1          ...............---------------
GAPDH_Pb1           ------------------------------
```
